# Comparison between asymptotic and re-randomisation tests under non-proportional hazards in a randomised controlled trial using the minimisation method

**DOI:** 10.1186/s12874-024-02295-2

**Published:** 2024-07-30

**Authors:** Ryusei Kimura, Shogo Nomura, Kengo Nagashima, Yasunori Sato

**Affiliations:** 1 Biostatistics Unit, Clinical and Translational Research Center, Keio University Hospital, Tokyo, 160-8582 Japan; 2 Graduate School of Health Management, Keio University, Tokyo, 252-0822 Japan; 3 Department of Biostatistics and Bioinformatics, Graduate School of Medicine, The University of Tokyo, Tokyo, 113-0033 Japan; 4 Department of Preventive Medicine and Public Health, Keio University School of Medicine, Tokyo, 160-8582 Japan

**Keywords:** Minimisation, Non-proportional hazards, Re-randomisation test, Weighted log-rank test, MaxCombo test, Restricted mean survival time, Survival analysis

## Abstract

**Background:**

Pocock-Simon’s minimisation method has been widely used to balance treatment assignments across prognostic factors in randomised controlled trials (RCTs). Previous studies focusing on the survival outcomes have demonstrated that the conservativeness of asymptotic tests without adjusting for stratification factors, as well as the inflated type I error rate of adjusted asymptotic tests conducted in a small sample of patients, can be relaxed using re-randomisation tests. Although several RCTs using minimisation have suggested the presence of non-proportional hazards (non-PH) effects, the application of re-randomisation tests has been limited to the log-rank test and Cox PH models, which may result in diminished statistical power when confronted with non-PH scenarios. To address this issue, we proposed two re-randomisation tests based on a maximum combination of weighted log-rank tests (MaxCombo test) and the difference in restricted mean survival time (dRMST) up to a fixed time point $\tau$, both of which can be extended to adjust for randomisation stratification factors.

**Methods:**

We compared the performance of asymptotic and re-randomisation tests using the MaxCombo test, dRMST, log-rank test, and Cox PH models, assuming various non-PH situations for RCTs using minimisation, with total sample sizes of 50, 100, and 500 at a 1:1 allocation ratio. We mainly considered null, and alternative scenarios featuring delayed, crossing, and diminishing treatment effects.

**Results:**

Across all examined null scenarios, re-randomisation tests maintained the type I error rates at the nominal level. Conversely, unadjusted asymptotic tests indicated excessive conservatism, while adjusted asymptotic tests in both the Cox PH models and dRMST indicated inflated type I error rates for total sample sizes of 50. The stratified MaxCombo-based re-randomisation test consistently exhibited robust power across all examined scenarios.

**Conclusions:**

The re-randomisation test is a useful alternative in non-PH situations for RCTs with minimisation using the stratified MaxCombo test, suggesting its robust power in various scenarios.

**Supplementary Information:**

The online version contains supplementary material available at 10.1186/s12874-024-02295-2.

## Background

Randomisation has been widely used to evaluate the efficacy and safety of interventions in clinical trials, ensuring comparability by achieving the balance for treatment assignments across prognostic factors. In randomised controlled trials (RCTs) with limited sample sizes and several prognostic factors, simple randomisation may not be sufficient to balance treatment assignments across prognostic factors. In such cases, stratified randomisation or Pocock–Simon’s minimisation method [1, 2] is often used. Stratified randomisation aims to balance the treatment assignments within each stratum; however, this objective becomes more challenging as the number of strata increases. Conversely, minimisation aims to achieve a marginal balance by sequentially assigning a new patient to the arm, which minimises the overall imbalance across the stratification factors. Consequently, RCTs that use minimisation are anticipated to have a higher number of strata relative to their sample sizes, necessitating careful consideration when choosing an analysis plan in these situations.

Previous studies have shown that a statistical test that relies on asymptotic normality without adjusting for the stratification factors used in minimisation is conservative [3–7]. Moreover, performing asymptotic tests with adjustment for all stratification factors may be unfeasible due to small or zero sample sizes within some strata. According to the FDA’s covariate adjustment guideline [8], “sponsors should discuss their proposal with the relevant review division if the number of covariates is large relative to the sample size or if proposing to adjust for a covariate with many levels.” In the survival analysis, adjusted Cox proportional hazards (PH) models exhibit an inflated type I error rate when the sample size is small [9]. Thus, regardless of the adjustment in the asymptotic test, the type I error rate may not be maintained at the nominal level. One potential solution is a re-randomisation test [10], which is expected to hold the type I error rate at the nominal level and improve power when the stratification factors are unadjusted in the test [11].

Several RCTs with minimisation have suggested the presence of non-PH treatment effects, which are typically classified as delayed, crossing, or diminishing. In an RCT with minimisation that compared the efficacy of pembrolizumab with that of a placebo in patients with completely resected stage III melanoma [12], non-PH effects were observed on recurrence-free survival. The results of this trial indicated a delayed effect in the overall population, where the survival probabilities of both groups remained similar for the first 3 months, and thereafter, the pembrolizumab group exhibited a higher survival probability than the placebo group. Moreover, a crossing effect was observed in a specific subgroup, where the pembrolizumab group initially demonstrated a lower survival probability than the placebo group; however, this trend was reversed in the later trial periods. Another study suggested a diminishing effect [13]. In these non-PH situations, the use of re-randomisation tests based on the log-rank test (LRT) and Cox PH models may reduce the statistical power, and the estimated hazard ratio (HR) may not be clinically interpretable [14, 15]. To the best of our knowledge, no previous study has evaluated the performance of re-randomisation tests under non-PH situations in RCTs using minimisation.

To address the limitations associated with non-PH situations, we proposed two re-randomisation tests based on statistics that do not rely on the PH assumption. First, we used a maximum combination of weighted LRT (WLRT) from the Fleming-Harrington (FH) $G^{\rho ,\gamma }$ class [16], known as the MaxCombo test [17–20]. This test demonstrates a robust higher power compared to the LRT under some non-PH scenarios. The concept of using re-randomisation to derive the null distribution for such a maximum combination was described by Ganju et al. [21, 22]. They demonstrated that the unadjusted MaxCombo-based re-randomisation test, in a setting with simple randomisation, exhibits a robust high power nearly equivalent to the highest power achieved by the WLRT among combinations $(G^{0,0},G^{1,0},G^{0,1})$. However, they did not evaluate tests with different methods, such as dRMST, or stratified WLRTs and stratified MaxCombo tests. Second, we used a restricted mean survival time (RMST), defined as the mean survival time up to a fixed time point $\tau$ [23]. The difference in RMST (dRMST) can be clinically interpretable as follows, even under non-PH situations: “How long, on average, the time to event onset can be extended when patients are followed up until the specific time point $\tau$.” [24] These tests can be extended to adjust for randomisation stratification factors by stratification and regression adjustments.

In this study, we aimed to evaluate the performance of re-randomisation tests based on the aforementioned statistics, assuming various non-PH situations for RCTs with minimisation. We compared these methods in terms of their empirical type I error rate and power using numerical simulations. “Methods” section provides an overview of the testing procedure for both the asymptotic and re-randomisation tests. In “Simulation study” section, we explained the simulation settings and presented the results; the results are discussed in “Discussion” section.

## Methods

We considered a two-armed comparison with a single survival primary endpoint in an RCT using minimisation that includes prognostic factors.

### Testing null hypothesis

We defined the test statistics corresponding to the LRT and Cox PH model, and for the proposed methods, the MaxCombo test and dRMST, denoted as $Z^{(\text {LRT})}$, $Z^{(\text {Cox})}$, $Z_{\text {max}}^{(\text {WLRT})}$ and $Z^{(\text {dRMST})}$, respectively. Furthermore, for these adjusted tests, we introduced $Z^{(\text {SLRT})}$, $Z^{(\text {ACox})}$, $Z_{\text {max}}^{(\text {SWLRT})}$, and $Z^{(\text {IPCW})}$. All of these *Z*-based test statistics asymptotically follow a standard normal distribution under the null hypothesis. The comprehensive details of each statistic are provided in Appendices A to D.

Let $S_1(t)$ and $S_2(t)$ be the survival functions of the experimental and control arms, respectively. Throughout this manuscript, we focused on one-sided tests to demonstrate the superiority of our experimental arm. The tests, including the LRT, WLRT, and MaxCombo test, were based on the null hypothesis $H_0$ and alternative hypothesis $H_1$:

$$\begin{aligned} & H_0: S_1(t)=S_2(t) \ \text {for all} \ t \ge 0, \\ & H_1: S_1(t)>S_2(t) \ \text {for some} \ t \ge 0. \end{aligned}$$

The $H_0$ was tested using $Z^{(\text {LRT})}$, $Z_{\text {max}}^{(\text {WLRT})}$, or $Z_{\text {max}}^{(\text {SWLRT})}$. We defined the RMST up to time point $\tau$ for each arm $g\ (=1,2)$ as $\mu _g(\tau )= \int _0^\tau S_g(t)dt$. For dRMST, the hypotheses were as follows:

$$\begin{aligned} H_0: \mu _1(\tau )-\mu _2(\tau )=0 \ \text {vs} \ H_1: \mu _1(\tau ) > \mu _2(\tau )=0, \end{aligned}$$

which was tested using $Z^{(\text {dRMST})}$.

For the regression-based method, both the Cox and dRMST models were based on the following hypotheses:

$$\begin{aligned} H_0: \beta _0=0 \ \text {vs} \ H_1: \beta _0 < 0, \end{aligned}$$

where $\beta _0$ represents the coefficient parameter for the treatment effect. We can test $H_0$ using $Z^{(\text {Cox})}$, $Z^{(\text {ACox})}$ or $Z^{(\text {IPCW})}$. The treatment effects $\beta _0$ were investigated rather than the other effects of the covariates.

Furthermore, we considered the strong null hypothesis [25, 26], which suggests that the survival probability in the experimental arm consistently remains less than that in the control arm, despite the hazard function initially favouring the control arm in the early trial periods:

$$\begin{aligned} & H_0^{\text {strong}}: S_1(t) \le S_2(t) \ \text {for all} \ t \ge 0, \\ & H_1^{\text {strong}}: S_1(t) > S_2(t) \ \text {for some} \ t \ge 0. \end{aligned}$$

Although the probability of falsely rejecting $H_0^{\text {strong}}$ is expected to be below the nominal level, one-sided WLRTs from $G^{0,1}$, $G^{1,1}$, and the associated MaxCombo test may exhibit an inflated type I error rate in the strong null scenario without a covariate [26, 27]. This is because events early in the experimental arm unfairly favour this arm for these tests [28]. We evaluated the type I error rates of MaxCombo tests in strong null scenarios that incorporated prognostic factors.

### Re-randomisation tests

When testing $H_0$ using a re-randomisation test, the treatment assignments are regenerated based on the actual randomisation procedure. During this regenerated treatment assignment process, the survival time, covariates, and order of patient entry remained fixed. Specifically, *M* datasets are generated to correspond with the observed dataset; these datasets include survival times and covariates identical to those in the observed dataset, along with regenerated treatment assignment sequences that may be identical to each other. For each iteration, we obtained the test statistics $S_m$ for $m=1,\ldots ,M$ through Monte Carlo simulations. Subsequently, using the approximated null distribution derived from these iterations, the one-sided *P*-value for this approach was calculated using the formula $\sum _{m=1}^{M}I\left( S_m \ge S_{\text {obs}}\right) /M$, where $S_{\text {obs}}$ represents the test statistic computed on the observed dataset [10].

Users can specify their preferred test statistics $S_m$ and $S_\text {obs}$ to test the corresponding $H_0$ as described in “Testing null hypothesis” section. The *P*-value for the MaxCombo-based asymptotic test is determined by the numerical integration of the multivariate normal distribution to account for multiplicity adjustment owing to the correlation among the four WLRT statistics (detailed in Appendix B). Conversely, for MaxCombo-based re-randomisation tests, $Z_{\text {max}}^{(\text {WLRT})}$ is directly adopted for both $S_m$ and $S_\text {obs}$, thereby regenerating the approximated null distribution of $Z_{\text {max}}^{(\text {WLRT})}$. We can apply the stratified MaxCombo test, $Z_{\text {max}}^{(\text {SWLRT})}$, analogously. The null distribution for the maximum combination statistics, $Z_{\text {max}}^{(\text {WLRT})}$ and $Z_{\text {max}}^{(\text {SWLRT})}$, can be derived from the re-randomisation as explained by Ganju et al. [21, 22]. Re-randomisation tests based on other tests, such as the LRT, Cox PH models, and dRMST, can also be constructed using the corresponding *Z*-based test statistics.

A numerical issue occurs in which $Z^{(\text {dRMST})}$ cannot be computed, due to censoring. This issue arises when the longest observed survival time in either arm is shorter than $\tau$ and is censored. Horiguchi et al. [29] illustrated this problem in detail and proposed several solutions. As their results indicated no differences between all evaluated methods, we simply adopted Method 2 [29], which extends the survival curve horizontally to $\tau$. Although this extrapolation-based approach was originally employed for null distributions during re-randomisation and not for observed data, they regenerated the observed data until a pre-specified number of simulations were reached. To reduce the simulation execution time, we applied Method 2 [29] to the observed data. Consequently, both $Z^{(\text {dRMST})}$ and $Z^{(\text {IPCW})}$ are computable, except when no events are observed in either arm. In these exceptional cases, neither $Z^{(\text {dRMST})}$ nor $Z^{(\text {IPCW})}$ can be computed owing to the failure to estimate their standard errors, even when using the method of Horiguchi et al.; thus, such cases were excluded from our simulation results.

## Simulation study

### Setup

To evaluate the performance of the aforementioned statistics in the asymptotic and re-randomisation tests, we calculated the empirical type I error rates and powers via numerical simulations, assuming two-armed RCTs with a 1:1 allocation ratio using minimisation. For the *i*th patient, the observed survival time was denoted as $T_i=\min (Y_i, C_i)$, where $T_i$ denotes an event if $Y_i \le C_i$, and otherwise, $T_i$ denotes right censoring. We assumed that the censoring time $C_i$ is independent of event time $Y_i$. Regarding prognostic factors, we set $Z_{i1}, Z_{i2} \overset{\text {iid}}{\sim } \text {Bernoulli}(2/3)$ and $Z_{i3} \sim \text {Bernoulli}(1/3)$. We generated $Y_i$ following a piecewise exponential distribution with the rate parameter $\lambda _{i}(t)$, which is modelled as follows:

$$\begin{aligned} \lambda _{i}(t)=\lambda _0 \exp \left[ \left\{ \beta _1 I(t<\epsilon ) + \beta _2 I(t \ge \epsilon ) \right\} Z_{i0}+\gamma _1 Z_{i1}+\gamma _2 Z_{i2}+\gamma _3 Z_{i3} \right] , \end{aligned}$$

where $I(\cdot )$ denotes an indicator function, $Z_{i0}$ is a treatment assignment based on minimisation with an assignment probability of 0.7. For all scenarios, we set covariate effects $\gamma _1, \gamma _2, \gamma _3$ as the common effect ($\gamma _1 = \gamma _2 = \gamma _3 = \gamma$), on the logarithm of the HR scale (log-HR). The treatment effects, $\beta _1$ and $\beta _2$, are on the log-HR scale and have been positioned before and after time point $\epsilon$ (months). The piecewise HR for treatment effects was different from the single HR from the Cox PH model. We assumed that a patient is uniformly accrued within 20 months and is followed up for at least 20 months; that is, $C_i$ follows a uniform distribution on $\left[ 20, 40 \right]$. The chosen total sample sizes, denoted by *n*, were 50, 100, and 500. All three prognostic factors were incorporated into the stratification using the minimisation scheme.

**Fig. 1:**
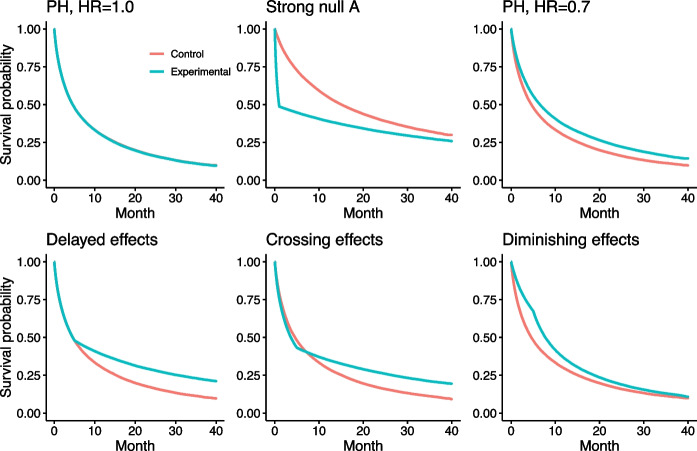
The marginal survival plots for each scenario

Initially, we considered two null scenarios: null scenarios with constant treatment effects ($\text {HR}=1.00$ over time) and a strong null scenario A, where the experimental survival probability consistently favours that of the control arm. In strong scenario A, the HR for the treatment effect was $\exp {(\beta _1)}=16.0$ in favour of the control arm for the initial month, subsequently shifting to $\exp {(\beta _2)}=0.8$ in favour of the experimental arm. In this scenario, the one-sided WLRTs from $G^{1,1}$, $G^{0,1}$ and the associated MaxCombo test, may result in a false rejection and advocate the alternative hypothesis that supports the experimental arm [26]. The parameter settings were based on the studies by Freidlin et al. [25] and Roychoudhury et al. [27] and were slightly modified to adapt to our RCT setting with prognostic factors. Subsequently, our simulation results from the strong null scenario A deviated from our expectations, leading to the introduction of an additional strong null scenario B. This scenario exhibited weaker prognostic factor effects compared to scenario A, ensuring that the survival distribution for each stratum remained almost consistent with the marginal distribution. The survival plots for each stratum under strong null scenarios A and B are shown in Figures S1 and S2. The rationale behind the parameter settings for scenario B is described in “Empirical type I error rate” section. For alternative hypotheses, three non-PH scenarios were examined: delayed, diminishing, and crossing treatment effects. The marginal survival plots for each scenario are displayed in Fig. 1. The parameter settings for each scenario are presented in Table 1.

**Table 1:** Parameter settings for each scenario

Scenario	$\lambda _0$	$\epsilon$ (month)	$\exp (\beta _1)$	$\exp (\beta _2)$	$\exp (\gamma )$	Censoring rates $(\%)$	
						Test	Cont
Null	2.00	0.00	1.00	1.00	0.20	14	14
Strong null A	0.60	1.00	16.0	0.80	0.20	30	36
Strong null B	0.04	1.00	16.0	0.70	0.90	30	38
PH	2.00	0.00	0.70	0.70	0.20	19	14
Delayed	2.00	5.00	1.00	0.40	0.20	26	14
Crossing	2.00	5.00	1.25	0.40	0.20	24	14
Diminishing	2.00	5.00	0.40	0.95	0.20	16	14

We evaluated the following tests: the MaxCombo test, with $(G^{0,0}, G^{1,0}, G^{1,1}, G^{0,1})$, and the dRMST with $\tau = 30$ (months). For comparative purposes, we also included the LRT and Wald tests based on the Cox PH models. In non-PH scenarios, using the Cox PH model, which is a model misspecification, may result in the loss of statistical power. The results of the other tests, including each WLRT from $G^{1,0}, G^{1,1}$, $G^{0,1}$, and the dRMST with $\tau = 20, 25$ (months), are presented in Table S1 and Figures S3 and S4. Furthermore, the performance of these tests was evaluated by adjusting the randomisation stratification factors using stratification and/or regression. We test $H_0$ at a one-sided significance level $\alpha =0.025$. For re-randomisation tests with $M=1,000$, we consistently used the *Z*-based test statistic for both $S_m$ and $S_\text {obs}$. As described in “Re-randomisation tests” section, cases in which $Z^{(\text {dRMST})}$ could not be computed due to censoring were addressed using Method 2 [29], which extends the survival curve horizontally to $\tau$. Simulations were performed using the R software with the “survival” [30], “survRM2” [31], and “nph” [32] packages to obtain *Z*-based test statistics. The numbers of repetitions were 10,000 for the null scenarios and 5,000 for the other scenarios, including strong null scenarios.

### Results

#### Empirical type I error rate

**Null scenarios:** Across all sample sizes ($n=50, 100, 500$), the empirical type I error rates for the re-randomisation tests were maintained at the nominal level $2.5\%$ (Table 2). The unadjusted asymptotic tests exhibited conservative type I error rates for larger sample sizes. The adjusted asymptotic tests in the LRT and MaxCombo maintained a type I error rate of $2.5\%$, whereas those in Cox and dRMST showed inflated type I error rates when a limited sample size was used ($n=50$). The detailed results of the other tests, including those of dRMST with different $\tau$, are presented in Table S1.

**Table 2:** Comparison of type I error rates under the null scenario

		*n* = 50	*n* = 100	*n* = 500
Method	Test	Type I error rate $(\%)$		
Asymp	LRT	1.03	0.76	0.39
	MCT	1.41	0.96	0.66
	Cox	0.96	0.74	0.37
	dRMST	1.30	0.95	0.40
	Stratified LRT	2.68	2.35	2.35
	Stratified MCT	2.46	2.34	2.45
	Adjusted Cox	**3.03**	2.48	2.40
	Adjusted dRMST	**3.55**	2.80	2.59
Re-rand	LRT	2.62	2.49	2.51
	MCT	2.59	2.41	2.40
	Cox	2.62	2.49	2.51
	dRMST	2.59	2.51	2.45
	Stratified LRT	2.39	2.22	2.15
	Stratified MCT	2.23	2.22	2.49
	Adjusted Cox	2.67	2.27	2.28
	Adjusted dRMST	2.58	2.40	2.40

*Abbreviations*: *Asymp *Asymptotic test, *Re-rand *Re-randomisation test, *LRT *Log-rank test, *MCT *MaxCombo test, *dRMST *Difference in RMST, *n *total sample sizes

The range of Monte Carlo SE for type I error rates is 0.06 to 0.19 (%). Bold values exceed 2 $\times$ Monte Carlo SE + 2.50

**Strong null scenarios:** For strong null scenarios, we showed the type I error rates for MaxCombo tests in Table 3. The type I error rates of MaxCombo were not consistently at $0\%$ across all sample sizes in both the asymptotic and re-randomisation tests, notably exceeding 2.5$\%$ at $n=500$. However, the type I error rates of the stratified MaxCombo ranged from 0 to 0.12$\%$.

Surprisingly, the trends in type I error rates for MaxCombo and stratified MaxCombo differed. This discrepancy between the MaxCombo and its stratified counterpart may be attributable to the differences in the calculation of the LR score, that is, regarding whether the calculation was performed marginally. Specifically, the stratum with $Z_{i1}=Z_{i2}=Z_{i3}=0$ diverges from the marginal strong null scenario, as illustrated in Figure S1. To validate these observations, we further investigated the strong null scenario B. In this scenario, each stratum did not deviate substantially from the marginal settings by incorporating the weaker effects of the prognostic factors compared to that in scenario A (Figure S2). Both the MaxCombo and stratified MaxCombo showed inflated type I error rates (MaxCombo:1.90–4.80$\%$ and stratified MaxCombo:1.46–4.30$\%$, Table 4). The results of the other tests in scenarios A and B are listed in Tables S2 and S3.

**Table 3:** Comparison of type I error rates under the strong null scenario A

		*n* = 50	*n* = 100	*n* = 500
Method	Test	Type I error rate $(\%)$		
Asymp	MCT	1.86	2.28	**8.28**
	Stratified MCT	0.10	0.04	0.00
Re-rand	MCT	**3.44**	**5.36**	**17.06**
	Stratified MCT	0.12	0.04	0.00

*Abbreviations: Asymp *Asymptotic test, *Re-rand *Re-randomisation test, *MCT *MaxCombo test, *n *total sample sizes

The maximum Monte Carlo SE for type I error rates (%) is 0.53. Bold values exceed 2 $\times$ Monte Carlo SE + 2.50

**Table 4:** Comparison of type I error rates under strong null scenario B

		*n* = 50	*n* = 100	*n* = 500
Method	Test	Type I error rate $(\%)$		
Asymp	MCT	2.12	2.32	**4.80**
	Stratified MCT	1.66	1.78	**3.96**
Re-rand	MCT	1.90	2.14	**4.72**
	Stratified MCT	1.46	1.78	**4.30**

*Abbreviations: Asymp* Asymptotic test, *Re-rand *Re-randomisation test, *MCT *MaxCombo test, *n* total sample sizes

The range of Monte Carlo SE for type I error rates is 0.16 to 0.30 $(\%)$. Bold values exceed 2 $\times$ Monte Carlo SE + 2.50

#### Empirical power

**Fig. 2:**
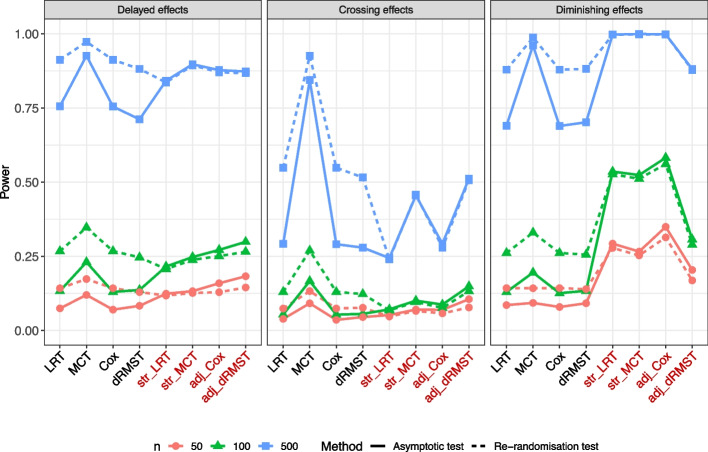
Comparison of powers under three different non-PH scenarios. Abbreviations: LRT, log-rank; MCT, MaxCombo; dRMST, difference in RMST; str, stratification; adj, adjustment through a regression model; *n*, total sample sizes

**Delayed effects scenarios:** The unadjusted re-randomisation tests indicated higher statistical powers than their corresponding asymptotic tests across all sample sizes (Fig. 2 on the left). In particular, among all tests, the MaxCombo-based re-randomisation test exhibited the highest power. Moving to the adjusted tests, re-randomisation tests in the LRT and MaxCombo indicated powers similar to those of their corresponding asymptotic tests across all sample sizes. In contrast, the adjusted re-randomisation tests in the Cox PH models and dRMST indicated slightly lower powers than their corresponding asymptotic tests at $n=50, 100$, with no substantial power differences at $n=500$. Among these adjusted tests, the MaxCombo test exhibited the highest power at $n=500$. For smaller sample sizes, $n=50, 100$, almost no difference was observed among the adjusted tests, except for the asymptotic tests in the Cox PH models and dRMST.

The conservatism of the unadjusted asymptotic tests and the slightly higher power of the adjusted Cox PH models and dRMST at $n=50, 100$ compared to their corresponding re-randomisation tests were consistently observed in subsequent scenarios. Therefore, we primarily focused on comparing the power of the re-randomisation tests in the following scenarios.

**Crossing effects scenarios:** Among the unadjusted re-randomisation tests, the MaxCombo-based re-randomisation test demonstrated a superior power, particularly at $n=500$ (Fig. 2 at the center). Among the adjusted re-randomisation tests, both dRMST-based and MaxCombo-based re-randomisation tests exhibited higher powers than the other adjusted re-randomisation tests, especially at $n=500$; no substantial power differences were observed among the adjusted re-randomisation tests at $n=50, 100$. The power of dRMST depended on the value of $\tau$ (Figure S3).

**Diminishing effect scenarios:** Similar to the crossing effects scenarios, among the unadjusted re-randomisation tests, the MaxCombo-based re-randomisation test demonstrated a higher power (Fig. 2 on the right). Among the adjusted re-randomisation tests, the powers of the Cox/dRMST were the highest and lowest at $n=50, 100$, respectively, with no substantial power differences at $n=500$.

**Proportional hazards scenarios:** As supplementary information, the results, which include all evaluated tests for the PH scenario, are presented in Figure S4. No substantial power differences were observed among the unadjusted re-randomisation tests, except for WLRT with $G^{(0,1)}$. Conversely, among the adjusted re-randomisation tests, the Cox PH models demonstrated higher power than the other tests, consistent with the findings reported by Xu et al. [9]. Following the Cox PH model, the LRT-based and MaxCombo-based re-randomisation tests showed the highest powers.

## Discussion

### Asymptotic tests versus re-randomisation tests

In the current study, we compared the performance of asymptotic and re-randomisation tests via numerical simulation, assuming various non-PH situations for RCTs with minimisation. As in previous studies [9, 33], the unadjusted asymptotic tests exhibited conservative type I error rates under null scenarios. Balancing treatment assignments by minimisation may lead to a correlation between treatment groups; thus, unadjusted tests that ignore this correlation yield conservative results. Accordingly, both the FDA guidelines and ICH E9 state the importance of accounting for the stratified randomisation factor in the analysis [8, 34]. Even with such adjustments, the Cox PH models and dRMST exhibited inflated type I error rates for $n=50$ owing to the small sample size. This finding suggests that the asymptotic tests adjusted for stratification factors do not always yield valid results. Regardless of covariate adjustment, the type I error rates of the re-randomisation tests remained at the nominal level across all examined sample sizes. Except for cases with inflated type I error rates, the re-randomisation tests preserved almost the same power as their asymptotic counterparts. Therefore, a re-randomisation test is a valuable alternative to an asymptotic test in RCTs with minimisation.

### The optimal re-randomisation test in terms of statistical power

Subsequently, we discussed which re-randomisation tests should be used, considering the aspect of power. Generally, the analysis used in RCTs must be pre-specified during the planning phase [34]. However, predicting the exact types of non-PH scenarios that may emerge is challenging, except in RCTs involving delayed effects in cancer immuno-oncology. Hence, a test that maintains robust power in various non-PH scenarios is required. Our simulation results show that the unadjusted re-randomisation tests in the LRT, Cox, and dRMST exhibited lower power than their corresponding adjusted re-randomisation tests. In contrast, the MaxCombo-based re-randomisation test demonstrated superior power under delayed and crossing effect scenarios, although it showed lower power than the other adjusted re-randomisation tests under diminishing effect scenarios. Among the adjusted re-randomisation tests, the LRT exhibited consistently lower powers across all examined non-PH scenarios. The adjusted Cox-based re-randomisation test showed the highest power under diminishing conditions, although it was inferior under the other non-PH scenarios. The adjusted dRMST-based re-randomisation test indicated a relatively high power for the crossing effect scenarios, but it was inferior in the other non-PH scenarios, with the power depending on $\tau$. Finally, the stratified MaxCombo-based re-randomisation tests exhibited consistently superior powers across all examined scenarios, including the PH scenarios. This observation suggests that, in terms of power, the stratified MaxCombo-based re-randomisation test is the optimal choice among examined tests.

### Considerations for the stratified MaxCombo-based re-randomisation test in RCTs using minimisation

Despite the promising performance of the stratified MaxCombo-based re-randomisation test, its application in RCTs warrants caution due to potential inflation of the type I error rate in strong null scenarios. This concern, discussed in the literature [35–40], is particularly relevant when considering the strict accuracy requirements of primary analyses in RCTs. We note that these studies focused on RCTs using simple randomisation, excluding stratified MaxCombo tests. In contrast, we demonstrated that, even under minimisation, the type I error rates of the MaxCombo tests (alternatively, the re-randomisation test) were inflated, except in some scenarios. However, given our assumption of using it in a non-primary analysis context, the MaxCombo-based re-randomisation test remains an attractive option, while recognising its limitations in such extreme scenarios.

Furthermore, we examined two types of strong null scenarios A and B, and found that in the strong null scenario A, stratified MaxCombo tests exhibited lower type I error rates compared to MaxCombo tests. In both scenarios, the MaxCombo test exhibited a type I error rate of up to 17$\%$. This result is consistent with the findings of a previous study [27]. In the strong null scenario A, the type I error inflation was not observed in the stratified MaxCombo test. This discrepancy arose because each stratum in scenario A deviated from the marginal setting (Figure S1 and S2). Under minimisation, several known prognostic factors with relatively strong effects exist, justifying the deviation of some strata from the marginal setting. Therefore, it is unrealistic to observe the inflation of the type I error rate for the stratified MaxCombo test under such an extreme null scenario in practical RCTs. This does not imply that the stratified MaxCombo test fundamentally overcomes this statistical flaw. However, even for those who do not accept the type I error rate inflation in the strong null scenario, it is worth considering the application of the stratified MaxCombo-based re-randomisation test as an option, possibly for non-primary analyses.

### Interpretability of estimands in non-proportional hazards scenarios

In non-PH cases, the interpretability of the estimands corresponding to the selected test is important. In particular, the HR estimated using the Cox PH model may not be clinically interpretable [14, 15]. Moreover, the estimated HR in non-PH scenarios varied depending on the study-specific censoring time distribution, such as accrual rate, accrual period, and follow-up period. Therefore, the estimated HR cannot be interpreted as a simple or meaningful weighted average of the time-specific HR in non-PH scenarios [41, 42]. As an alternative, a piecewise HR, which describes the change in treatment effect over time, or a weighted HR corresponding to the maximum WLRT within the MaxCombo test, may be useful to capture the characteristics of the non-PH situation [20, 27, 43]. However, even these estimands may remain subject to criticism when interpreting the treatment effect causally [14, 44]. Shifting focus from HRs, the dRMST has a 1:1 correspondence between the testing and estimand, which is clinically interpretable even under non-PH scenarios. In crossing effects scenarios, where interpreting the HR becomes particularly challenging, the adjusted dRMST-based re-randomisation tests showed relatively high power. Although the power of dRMST depends on $\tau$, considering that $\tau$ is typically selected from a clinical perspective, the dRMST-based re-randomisation test may be an attractive choice in terms of interpretability of the estimand. Importantly, in non-PH contexts, relying on a single summary measure and using test results alone to infer treatment efficacy is inadequate; thus, reporting multiple summary measures is recommended for a more comprehensive assessment of the treatment effect [27].

## Conclusion

Re-randomisation tests have emerged as a credible and methodologically robust alternative to asymptotic tests in RCTs employing minimisation, particularly under non-PH conditions. The efficacy of the adjusted dRMST-based re-randomisation test is scenario-specific and significantly influenced by the strategic selection of the time point $\tau$. In contrast, the stratified MaxCombo-based re-randomisation test has consistently demonstrated its pre-eminence in power across a broad spectrum of scenarios. Although whether an inflated type I error rate for the stratified MaxCombo-based re-randomisation test in the strong null scenario should be strictly controlled is debatable, this inflation is considerably reduced under some strong null scenarios for RCTs with minimisation. Consequently, considering the necessity of pre-specifying statistical analyses in RCT design, the stratified MaxCombo-based re-randomisation test is recommended for its steadfast and superior power in non-primary analysis.

### Supplementary Information

Additional file 1. Survival plots for each stratum under strong null scenarios are illustrated in Figure S1 and S2; simulation results under the null scenarios are provided in Table S1; results under the strong null scenarios for other tests not shown in the manuscript are provided in Tables S2 and S3; results under the non-PH scenarios for other tests not shown in the manuscript are illustrated in Figure S3; results under the PH scenarios are provided in Figure S4.

## Data Availability

The datasets used and/or analysed in the current study are available from the corresponding author upon reasonable request.

## Appendix

### A Weighted log-rank test and its stratification

We initially explained the WLRT based on the FH $G^{\rho ,\gamma }$ class [16]. Then, the stratified WLRT (SWLRT) can be naturally constructed by replacing the LR score for each stratum with the WLR score. Let $t_j\ (j=1,2,\ldots ,D)$ be the *j*th ordered event time, $d_{gj}$ be the number of events at $t_j$ in arm $g\ (=1,2)$, and $n_{gj}$ be the number of patients at risk of the event at $t_j$ in arm *g*. The WLR score is obtained using the following equation:

$$\begin{aligned} U^{\text {(WLRT)}}=\sum \limits _{j=1}^{D}w_j\left( d_{1j}-\frac{n_{1j}d_j}{n_j}\right) , \end{aligned}$$

where $d_{j} = d_{1j}+d_{2j}$ and $n_{j} = n_{1j}+n_{2j}$. The variance of $d_{1j}$ is given by:

$$\begin{aligned} v_{1j}=w_j^2 \frac{n_{1j}n_{2j}d_{j}(n_{j}-d_{j})}{n_j^{2}(n_{j}-1)}. \end{aligned}$$

Subsequently, the variance of $U^{\text {(WLRT)}}$ is $\hat{V} \left[ U^{\text {(WLRT)}}\right] =\sum _{j=1}^{D}v_{1j}$. Therefore, the statistic for WLRT is calculated as follows: $Z^{\text {(WLRT)}}=\frac{U^{\text {(WLRT)}}}{\sqrt{\hat{V}\left[ U^{\text {(WLRT)}}\right] }}$, which asymptotically follows a standard normal distribution under the null hypothesis [45].

For the stratification analysis, we extended the indices to account for the stratum $s\ (=1,2,\ldots ,S)$. Hence, $n_{gjs}$ and $d_{gjs}$ represent the number at risk and the number of events in stratum *s*, respectively. The WLR score for each stratum is calculated as follows:

$$\begin{aligned} U_s^{\text {(WLRT)}}=\sum \limits _{j=1}^{D}w_j\left( d_{1js}-\frac{n_{1js}d_{js}}{n_{js}}\right) , \end{aligned}$$

where $d_{js} = d_{1js}+d_{2js}$ and $n_{js} = n_{1js}+n_{2js}$. The variance of $d_{1js}$ is calculated as follows:

$$\begin{aligned} v_{1js}=w_j^2 \frac{n_{1js}n_{2js}d_{js}(n_{js}-d_{js})}{n_{js}^{2}(n_{js}-1)}. \end{aligned}$$

Subsequently, the variance of $U_s^{\text {(WLRT)}}$ is calculated as follows: $\hat{V}\left[ U_s^{\text {(WLRT)}}\right] =\sum _{j=1}^{D}v_{1js}$. Therefore, the test statistic for SWLRT is defined as follows:

$$\begin{aligned} Z^{\text {(SWLRT)}}=\frac{\sum _{s=1}^{S}U_s^{(\text {WLRT})}}{\sqrt{\sum _{s=1}^{S}\hat{V}\left[ U_s^{\text {(WLRT)}}\right] }}, \end{aligned}$$

which asymptotically follow a standard normal distribution under the null hypothesis [45].

The weight function for the $G^{\rho ,\gamma }$ class is defined as $w_{j}=\left( \hat{S}(t_{j-1})\right) ^{\rho }\left( 1-\hat{S}(t_{j-1})\right) ^{\gamma }$, where $\rho \ge 0, \gamma \ge 0$, and $\hat{S}(t_{j-1})$ denote the pooled KM estimator. The representative FH classes, $G^{1,0}$, $G^{1,1}$, and $G^{0,1}$ are weighted to the early, middle, and late events, respectively, whereas $G^{0,0}$ corresponds to the LRT. The WLRT with unfavourable weights that do not align with the observed data may exhibit a reduced power [20]. If the non-PH type, such as delayed, crossing, or diminishing treatment effects, is unpredictable, a robust method for non-PH types may be desirable.

### B MaxCombo test and its stratification

A maximum combination of WLRT statistics, known as the MaxCombo test, demonstrates a robust higher power compared to LRT under a non-PH situation [17–20]. The MaxCombo for one-sided test is defined as follows:

$$\begin{aligned} Z_{\text {max}}^{(\text {WLRT})}=\max {\left( Z_1^{(\text {WLRT})},Z_2^{(\text {WLRT})},\ldots ,Z_L^{(\text {WLRT})}\right) }, \end{aligned}$$

where $Z_l^{(\text {WLRT})}$ ($l=1,2,\ldots ,L$) denotes some classes of the WLRT. Among several combinations of $Z_l^{(\text {WLRT})}$, Lin et al. [20] proposed the MaxCombo test based on $Z_{\text {max}}^{(\text {WLRT})}$ with $Z_1^{(\text {WLRT})}, Z_2^{(\text {WLRT})}, Z_3^{(\text {WLRT})}$, and $Z_4^{(\text {WLRT})}$ corresponding to $G^{0,0}, G^{1,0}, G^{1,1}$, and $G^{0,1}$. The $Z_{\text {max}}^{(\text {WLRT})}$ asymptotically follows a multivariate normal distribution with means of zero and the correlation matrix $\varvec{R}$ under the null hypothesis [19, 20]. Let the covariances of $G^{\rho _i,\gamma _i}$ and $G^{\rho _j,\gamma _j}$ be $\text {Cov}[G^{\rho _i,\gamma _i}, G^{\rho _j,\gamma _j}]$ and let the (*i*, *j*)-component $r_{ij}$ of $\varvec{R}$ be given by

$$\begin{aligned} r_{ij}= \left\{ \begin{array}{ll} \frac{\text {Cov}[G^{\rho _i,\gamma _i}, G^{\rho _j,\gamma _j}]}{\sqrt{V[G^{\rho _i,\gamma _i}] V[G^{\rho _j,\gamma _j}]}} = \frac{V\left[ G^{\frac{\rho _i+\rho _j}{2}, \frac{\gamma _i+\gamma _j}{2}}\right] }{\sqrt{V[G^{\rho _i,\gamma _i}] V[G^{\rho _j,\gamma _j}]}} & \text {for} \ i\ne j\\ 1 & \text {for} \ i=j, \end{array}\right. \end{aligned}$$

as described previously  [19, 20, 27]. The *P*-value of the MaxCombo test was calculated by numerical integration based on the above multivariate normal distribution with using the algorithm proposed by Genz [46]. For the adjustment of covariates, the stratified MaxCombo test statistics, $Z_{\text {max}}^{(\text {SWLRT})}$ can be naturally constructed by replacing $Z_l^{(\text {WLRT})}$ with $Z_l^{(\text {SWLRT})}$.

### C Cox proportional hazards model

The Cox PH model [47] is routinely used to estimate the HR as a treatment effect under the PH assumption. Let $\varvec{Z}_{i}$ be a $p+1$-dimensional covariate vector comprising a treatment group indicator and *p* covariates. For multivariable analysis, the Cox PH model is expressed as follows:

$$\begin{aligned} h(\varvec{Z}_i, t)=\exp (\varvec{\beta }^{\top }\varvec{Z}_i)h_0(t), \end{aligned}$$

where $h(\varvec{Z}_i, t)$ represents the hazard function for the *i*th patient at time *t*; vector $\varvec{\beta }^{\top }=(\beta _0,\ldots ,\beta _p)$ is a $p+1$-dimensional regression coefficients, including a treatment effect $\beta _0$; and $h_0(t)$ is a baseline hazard function for time *t*.

Moreover, the stratified Cox PH model is expressed using the following equation:

$$\begin{aligned} h_s(Z_{i}, t)=\exp (\beta _0^*Z_{i})h_{0s}(t), \end{aligned}$$

where $h_s(Z_{i}, t)$ represents the hazard function for *i*th patient at time *t* in stratum $s~(=1,\ldots ,S)$, $Z_{i}$ is the treatment group indicator for *i*th patient, $\beta _0^*$ is the coefficient for the treatment group indicator, and $h_{0s}(t)$ is the baseline hazard function for time *t* in stratum *s*.

The estimation of $\varvec{\beta }$ and $\beta ^*_0$ are based on a partial likelihood [45]. Given our interest in treatment effects, Wald tests based on the aforementioned equations are expressed as follows: $Z^{(\text {ACox})}=\frac{\hat{\beta }_0}{\sqrt{\hat{V} [\hat{\beta }_0]}}$ and $Z^{(\text {SCox})}=\frac{\hat{\beta }^*_0}{\sqrt{\hat{V} [\hat{\beta }^*_0]}}$, respectively. For univariate situations with $p=0$, we denote the test statistics by $Z^{(\text {Cox})}$. These test statistics asymptotically follow a standard normal distribution under the null hypothesis [45].

### D Difference in RMST and its adjustment

The RMST is defined as the mean survival time up to a fixed time point $\tau$ [23]. Let $D_g(\tau )$ be the total number of events up to time point $\tau$ in arm $g\ (=1,2)$ and $\hat{S}_g(t)$ be a KM estimator of $S_g(t)$. For each arm, the RMST estimated by the direct integration of the KM curve method [45, 48] is calculated as follows:

$$\begin{aligned} \hat{\mu }_g(\tau )= \int _{0}^{\tau }\hat{S}_g(t)dt=\sum \limits _{j=0}^{D_g(\tau )}(t_{j+1}-t_j)\hat{S}_g(t_j), \end{aligned}$$

where $t_0=0$ and $t_{D_g(\tau )+1}=\tau$. The variance of $\hat{\mu }_g(\tau )$ based on Greenwood’s formula [49] is calculated as follows:

$$\begin{aligned} \hat{V}[\hat{\mu }_g(\tau )]=\sum \limits _{j=1}^{D_g(\tau )}\left[ \sum \limits _{k=j}^{D_g(\tau )}(t_{k+1}-t_k) \hat{S}_g(t_k) \right] ^2 \frac{d_{gj}}{n_{gj}(n_{gj}-d_{gj})}. \end{aligned}$$

Thus, the test statistics for the dRMST were constructed as

$$\begin{aligned} Z^{(\text {dRMST})}=\frac{\hat{\mu }_1(\tau )-\hat{\mu }_2(\tau )}{\sqrt{\hat{V}[\hat{\mu }_1(\tau )]+\hat{V}[\hat{\mu }_2(\tau )]}}, \end{aligned}$$

which asymptotically follows the standard normal distribution under the null hypothesis [48].

For covariate adjustments, the inverse probability of censoring weighted (IPCW) method was proposed by Tian et al. [50]. Let $C_i\ (i=1,\ldots ,n)$ be a non-negative random variable denoting the censoring time for the *i*th patient, and $\eta (\cdot )$ be a general link function. The covariate vector $\varvec{\beta }$, which includes a treatment effect $\beta _0$, can be estimated using the estimating equation for the IPCW, based on $\eta (E[Y_{i}(\tau )\mid \varvec{X}_i])=\varvec{\beta }^{\top }\varvec{X}_i$, where $\varvec{X}_i^{\top }=(1,\varvec{Z}_{i}^{\top })$. The estimating equation for the IPCW is obtained as follows:

$$\begin{aligned} U^{(\text {IPCW})}(\varvec{\beta })=\frac{1}{n}\sum \limits _{j=1}^{D}\frac{1}{\hat{G}_{g_{j}}(t_{j}(\tau ))}\varvec{X}_j\{t_j(\tau )-\eta ^{-1}(\varvec{\beta }^{\top }\varvec{X}_j)\}, \end{aligned}$$

where $\hat{G}_g(\cdot )$ represents the KM estimators of $C_i$ in arm $g_j\ (=1,0)$ for the patient who experienced the *j*th event, and $t_j(\tau ) = \min {(t_j,\tau )}$ denotes the restricted time to event. The variance of $\hat{\varvec{\beta }}$ can be estimated using a sandwich estimator. Hence, an IPCW-based test statistic is constructed using $Z^{(\text {IPCW})}=\frac{\hat{\beta }_0}{\sqrt{\hat{V}(\hat{\beta }_0)}}$, which asymptotically follows a standard normal distribution under the null hypothesis.

## Abbreviations

RCT: Randomised controlled trial
PH: Proportional hazards
KM: Kaplan–Meier
FH: Fleming–Harrington
LRT: Log-rank test
WLRT: Weighted log-rank test
SWLRT: Stratified weighted log-rank test
dRMST: difference in restricted mean survival time
IPCW: Inverse probability of censoring weighted
