# Supplementary material for: Comparison between asymptotic and re-randomisation tests under non-proportional hazards in a randomised controlled trial using the minimisation method
Source: BMC Med Res Methodol. 2024 Jul 30;24:166. doi: 10.1186/s12874-024-02295-2 (PMC11290221; doi:10.1186/s12874-024-02295-2)
Supplement: Supplementary file 1 — Additional file 1. Survival plots for each stratum under strong null scenarios are illustrated in Figure S1 and S2; simulation results under the null scenarios are provided in Table S1; results under the strong null scenarios for other tests not shown in the manuscript are provided in Tables S2 and S3; results under the non-PH scenarios for other tests not shown in the manuscript are illustrated in Figure S3; results under the PH scenarios are provided in Figure S4. [file 12874_2024_2295_MOESM1_ESM.pdf]

Additional file 1 for "Comparison between asymptotic and re-randomisation tests under non-proportional hazards in a randomised controlled trial using the minimisation method"

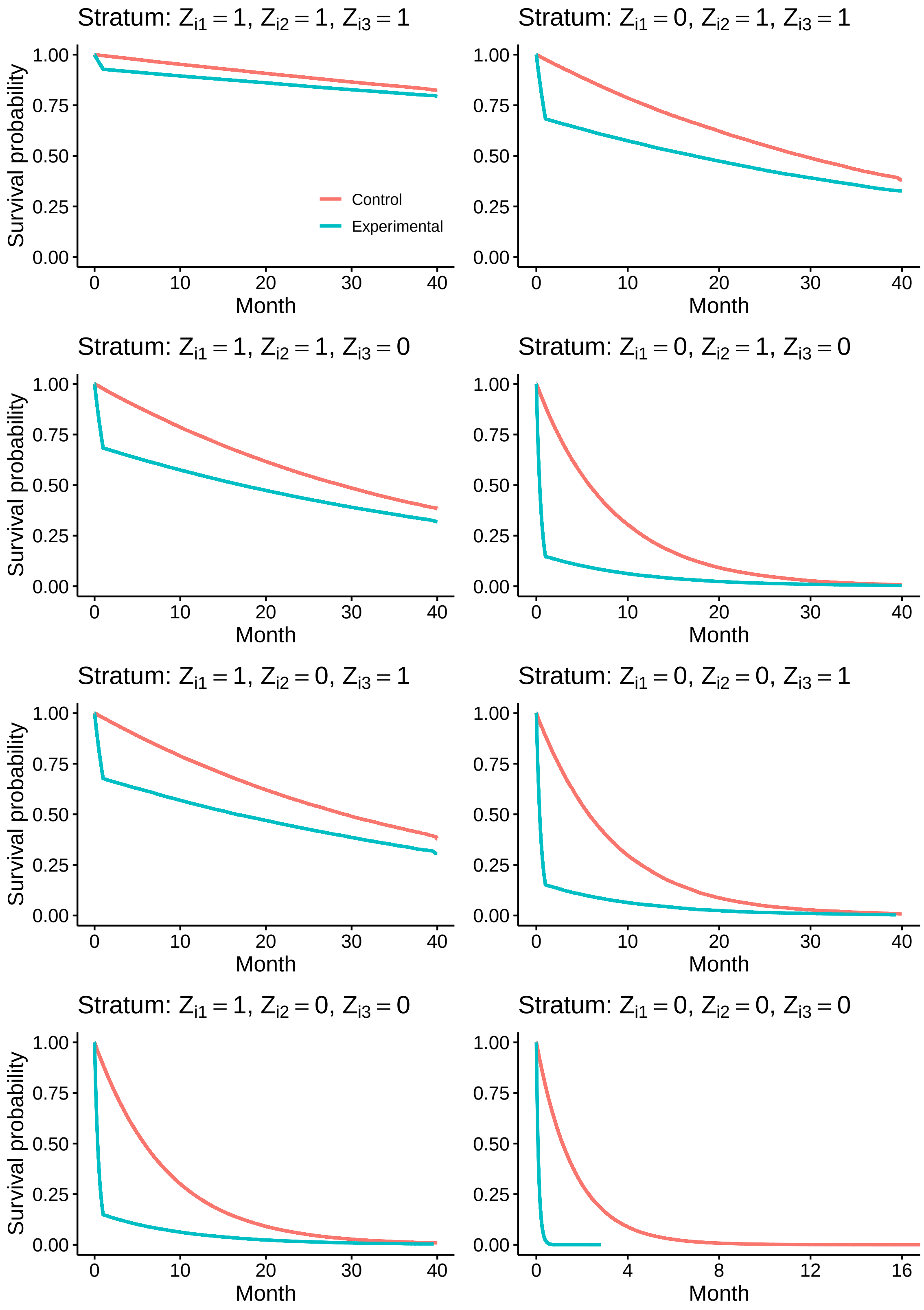

Figure S1: Survival plots for each stratum under the strong null scenario A

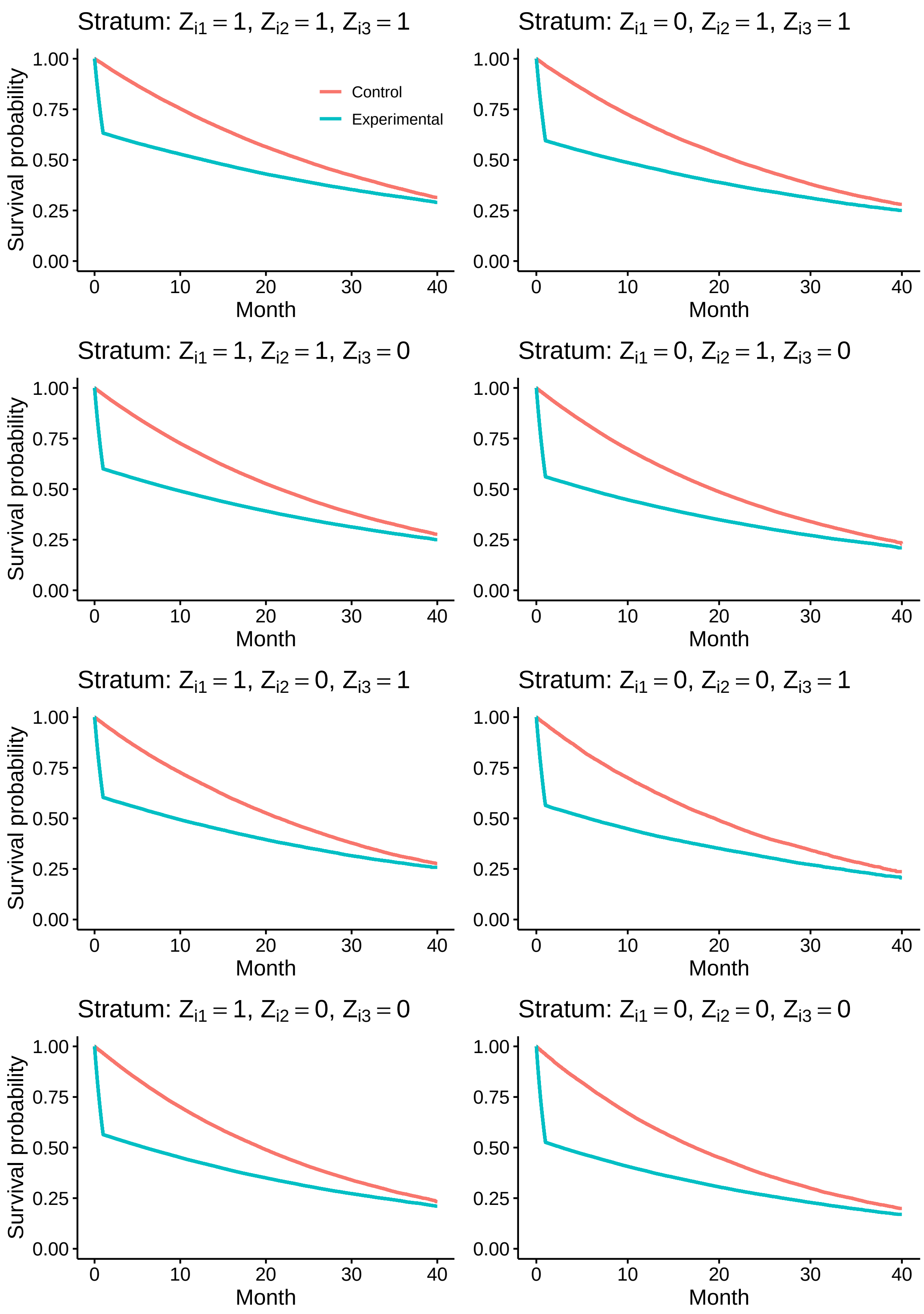

Figure S2: Survival plots for each stratum under the strong null scenario B

Table S1: Comparison of type I error rates under the null scenario

		$n = 50$	$n = 100$	$n = 500$
Method	Test	Type I error rate (%)		
Aymp	WLRT10	0.97	0.57	0.27
	WLRT11	1.45	1.23	0.69
	WLRT01	2.18	1.42	1.33
	MCT	1.41	0.96	0.66
	dRMST20	1.23	0.91	0.38
	dRMST25	1.20	1.00	0.40
	dRMST30	1.30	0.95	0.40
	Stratified WLRT10	2.45	2.31	2.13
	Stratified WLRT11	2.38	2.42	2.58
	Stratified WLRT01	2.64	2.37	2.48
	Stratified MCT	2.46	2.34	2.45
	Stratified Cox	2.58	2.35	2.42
	Adjusted dRMST20	**3.52**	**3.01**	2.49
	Adjusted dRMST25	**3.65**	**2.88**	2.57
	Adjusted dRMST30	**3.55**	2.80	2.59
Re-rand	WLRT10	2.51	2.46	2.52
	WLRT11	2.67	2.24	2.36
	WLRT01	2.63	2.29	2.35
	MCT	2.59	2.41	2.40
	dRMST20	2.39	2.54	2.34
	dRMST25	2.53	2.46	2.38
	dRMST30	2.59	2.51	2.45
	Stratified WLRT10	2.39	2.24	2.14
	Stratified WLRT11	2.18	2.29	2.52
	Stratified WLRT01	2.29	2.09	2.40
	Stratified MCT	2.23	2.22	2.49
	Stratified Cox	2.52	2.29	2.35
	Adjusted dRMST20	2.41	2.57	2.51
	Adjusted dRMST25	2.69	2.45	2.47
	Adjusted dRMST30	2.58	2.40	2.40

Abbreviations: Asymp, asymptotic test; Re-rand, re-randomisation test; LRT, log-rank test; WLRT, weighted LRT (e.g., WLRT10 with $G^{1,0}$, WLRT11 with $G^{1,1}$, WLRT01 with $G^{0,1}$); MCT, MaxCombo test; dRMST, difference in RMST (e.g., dRMST20 with $\tau = 20$, dRMST20 with $\tau = 25$, dRMST30 with $\tau = 30$); n, total sample sizes

The range of Monte Carlo SE for type I error rates is 0.05 to 0.19 (%). Bold values exceed $2 \times$ Monte Carlo SE $+ 2.50$.

Table S2: Comparison of type I error rates under the strong null scenario A

Method	Test	$n = 50$	$n = 100$	$n = 500$
		Type I error rate (%)		
Asymp	LRT	0.00	0.00	0.00
	WLRT10	0.00	0.00	0.00
	WLRT11	1.28	0.78	0.30
	WLRT01	**3.46**	**4.54**	**13.62**
	Cox	0.00	0.00	0.00
	dRMST20	0.00	0.00	0.00
	dRMST25	0.00	0.00	0.00
	dRMST30	0.00	0.00	0.00
	Stratified LRT	0.00	0.00	0.00
	Stratified WLRT10	0.00	0.00	0.00
	Stratified WLRT11	0.06	0.02	0.00
	Stratified WLRT01	0.22	0.06	0.00
	Stratified Cox	0.00	0.00	0.00
	Adjusted Cox	0.00	0.00	0.00
	Adjusted dRMST20	0.00	0.00	0.00
	Adjusted dRMST25	0.00	0.00	0.00
	Adjusted dRMST30	0.04	0.00	0.00
Re-rand	LRT	0.06	0.02	0.00
	WLRT10	0.00	0.00	0.00
	WLRT11	2.22	1.90	0.92
	WLRT01	**5.32**	**7.30**	**21.96**
	Cox	0.06	0.02	0.00
	dRMST20	0.00	0.00	0.00
	dRMST25	0.00	0.00	0.00
	dRMST30	0.04	0.00	0.00
	Stratified LRT	0.00	0.00	0.00
	Stratified WLRT10	0.00	0.00	0.00
	Stratified WLRT11	0.08	0.02	0.00
	Stratified WLRT01	0.22	0.08	0.00
	Stratified Cox	0.00	0.00	0.00
	Adjusted Cox	0.00	0.00	0.00
	Adjusted dRMST20	0.00	0.00	0.00
	Adjusted dRMST25	0.00	0.00	0.00
	Adjusted dRMST30	0.02	0.00	0.00

Abbreviations: Asymp, asymptotic test; Re-rand, re-randomisation test; WLRT, weighted log-rank test (e.g., WLRT10 with $G^{1,0}$, WLRT11 with $G^{1,1}$, WLRT01 with $G^{0,1}$); dRMST, difference in RMST (e.g., dRMST20 with $\tau = 20$, dRMST20 with $\tau = 25$, dRMST30 with $\tau = 30$); n, total sample sizes

The maximum Monte Carlo SE for type I error rates is 0.59 (%).

Bold values exceed 2 $\times$ Monte Carlo SE + 2.50.

Table S3: Comparison of type I error rates under the strong null scenario B

		$n = 50$	$n = 100$	$n = 500$
Method	Test	Type I error rate (%)		
Asymp	LRT	0.06	0.00	0.00
	WLRT10	0.02	0.00	0.00
	WLRT11	2.08	1.44	0.88
	WLRT01	**4.12**	**4.18**	**7.98**
	Cox	0.06	0.00	0.00
	dRMST20	0.00	0.00	0.00
	dRMST25	0.00	0.00	0.00
	dRMST30	0.02	0.00	0.00
	Stratified LRT	0.08	0.00	0.00
	Stratified WLRT10	0.04	0.00	0.00
	Stratified WLRT11	2.18	1.48	0.74
	Stratified WLRT01	2.88	**3.44**	**7.00**
	Stratified Cox	0.04	0.00	0.00
	Adjusted Cox	0.06	0.00	0.00
	Adjusted dRMST20	0.00	0.00	0.00
	Adjusted dRMST25	0.00	0.00	0.00
	Adjusted dRMST30	0.02	0.00	0.00
Re-rand	LRT	0.04	0.00	0.00
	WLRT10	0.02	0.00	0.00
	WLRT11	1.98	1.48	0.84
	WLRT01	**3.64**	**4.06**	**8.08**
	Cox	0.04	0.00	0.00
	dRMST20	0.00	0.00	0.00
	dRMST25	0.00	0.00	0.00
	dRMST30	0.00	0.00	0.00
	Stratified LRT	0.10	0.00	0.00
	Stratified WLRT10	0.02	0.00	0.00
	Stratified WLRT11	1.86	1.62	0.68
	Stratified WLRT01	2.60	**3.26**	**7.12**
	Stratified Cox	0.04	0.00	0.00
	Adjusted Cox	0.04	0.00	0.00
	Adjusted dRMST20	0.00	0.00	0.00
	Adjusted dRMST25	0.00	0.00	0.00
	Adjusted dRMST30	0.00	0.00	0.00

Abbreviations: Asymp, asymptotic test; Re-rand, re-randomisation test; WLRT, weighted log-rank test (e.g., WLRT10 with $G^{1,0}$, WLRT11 with $G^{1,1}$, WLRT01 with $G^{0,1}$); dRMST, difference in RMST (e.g., dRMST20 with $\tau = 20$, dRMST20 with $\tau = 25$, dRMST30 with $\tau = 30$); n, total sample sizes

The maximum Monte Carlo SE for type I error rates is 0.39 (%).

Bold values exceed 2 × Monte Carlo SE + 2.50.

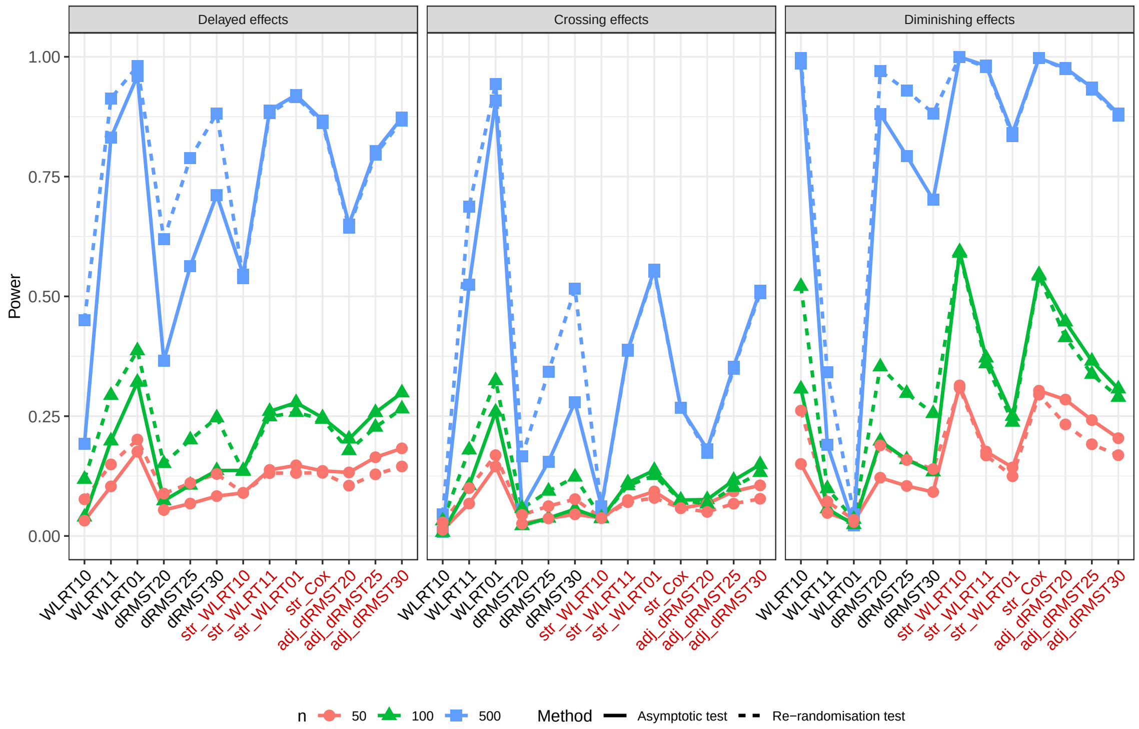

Figure S3: Comparison of powers under non-PH scenarios. Abbreviations: LRT, log-rank test; WLRT, weighted LRT (e.g., WLRT10 with $G^{1,0}$, WLRT11 with $G^{1,1}$, WLRT01 with $G^{0,1}$); dRMST, difference in RMST (e.g., dRMST20 with $\tau = 20$, dRMST20 with $\tau = 25$, dRMST30 with $\tau = 30$); str, stratification; adj, adjustment through a regression model; n, total sample sizes

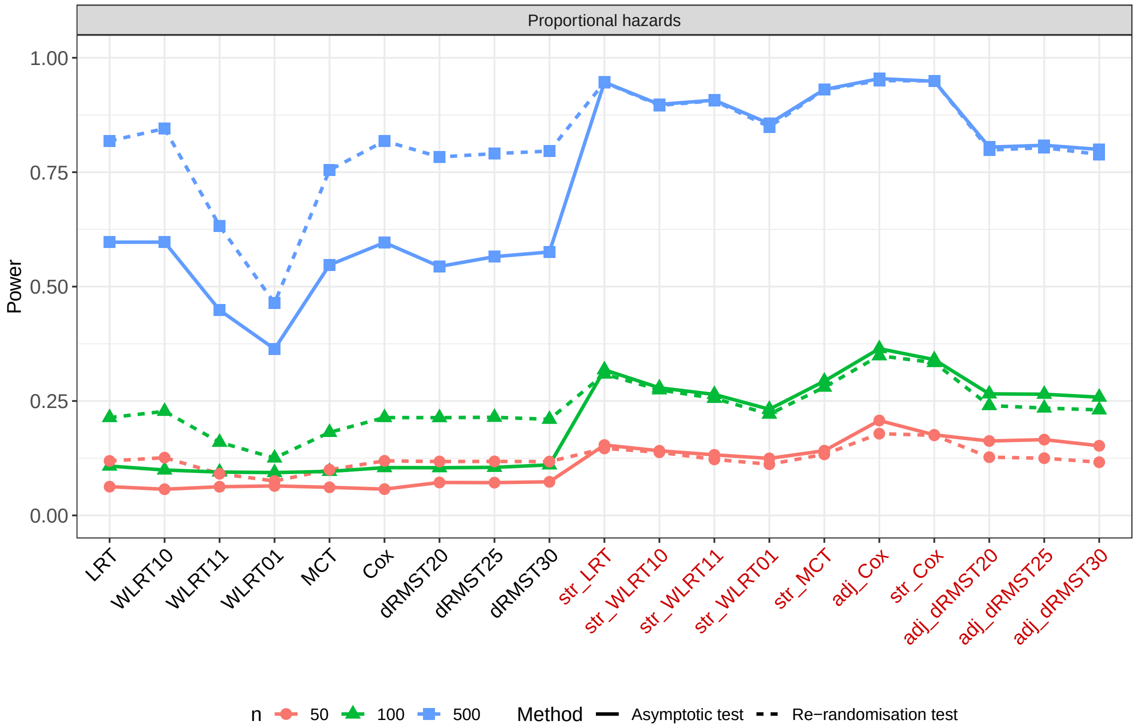

Figure S4: Comparison of powers under the PH scenario. Abbreviations: LRT, log-rank test; WLRT, weighted LRT (e.g., WLRT10 with $G^{1,0}$, WLRT11 with $G^{1,1}$, WLRT01 with $G^{0,1}$); MCT, MaxCombo test; dRMST, difference in RMST (e.g., dRMST20 with $\tau = 20$, dRMST20 with $\tau = 25$, dRMST30 with $\tau = 30$); str, stratification; adj, adjustment through a regression model; n, total sample sizes
